# First Restoration Experiment for Endemic *Fucus virsoides* on the Western Istrian Coast—Is It Feasible?

**DOI:** 10.3390/plants12071445

**Published:** 2023-03-24

**Authors:** Edi Gljušćić, Andrea Bilajac, Shannen Maree Smith, Mirjana Najdek, Ljiljana Iveša

**Affiliations:** Center for Marine Research, Ruđer Bošković Institute, G. Paliaga 5, 52210 Rovinj, Croatia

**Keywords:** *Fucus virsoides*, Northern Adriatic, ex situ cultivation, restoration

## Abstract

*Fucus virsoides* is an endemic species of the Mediterranean limited to the Adriatic Sea. In recent decades, it has undergone a severe regression, which is well documented in the northern Adriatic. To develop a tool for mitigating this problem, we tested the feasibility of *F. virsoides* restoration and designed a very simple yet effective method for ex situ cultivation and planting. We also tested the effect of positioning in the upper vs. lower intertidal on the growth of *F. virsoides.* After planting, the algae reached fertility in nine months, which was followed by a period of stagnation and reduction in size due to grazing and fouling. There were some differences in growth of the algae according to positioning in the intertidal at different measurement times, but that had little impact on the overall success of the restoration experiment. This represents, to our knowledge, the first successful *F. virsoides* ex situ cultivation and restoration attempt.

## 1. Introduction

*Fucus virsoides* J. Agardh (Fucales, Phaeophyceae) is a species endemic to the Mediterranean, only found in its coldest part, the Adriatic Sea [1]. It is one of 10 species of its genus, as well as the only one inhabiting the Mediterranean basin [2]. This species is limited to the intertidal zone of the Adriatic sea and is a functional equivalent to the much larger *Fucus* species that form substantial macroalgal forests in the intertidal zone of the oceanic coasts, as well as the Baltic and North seas [2]. While currently distributed mostly on the rocky eastern coast, from Venice, Italy, to Albania, the species has historically been found in Ancona on the western coast until the 1960s, when it went extinct [3,4,5,6,7,8,9,10]. Although considered locally ubiquitous in the past, especially in the northern Adriatic, nowadays it can be regarded as functionally extinct due to a small number of isolated remnant settlements. *F. virsoides* settlements in their original size were able to support a noticeable biodiversity and biomass of smaller algae and animal species, although this was explored mostly during the mid-20th century, but this was also touched upon later during the 2010s, albeit on a small scale due to already severely degraded settlements [3,4,11,12,13,14].

The species was considered to be very abundant and common on the Istrian coastline (northern Adriatic Sea), especially on the western coast, confirmed by floristic studies and herbarium collections from the 19th and early 20th century [3,15,16,17,18]. Large abundances were also measured during the late 1960s and early 1970s in the vicinity of Rovinj, with up to 5 kg/m^2^ (218 g/400 cm^2^) measured in Rovinj at the time [5,11]. Later, wet weight assessments were smaller, at 2.6–3.5 kg/m^2^ (107–140 g/400 cm^2^) in 1999 and 0.5–2.5 kg/m^2^ (20–100 g/400 cm^2^) in 2014, respectively [13,19]. Surprisingly, despite the perceived ordinariness, save for the settlements around Rovinj, almost no additional settlements were surveyed and mapped until the start of 21st century. This has, unfortunately, left a major gap in the distributional data for this species, which can cause serious obstacles in conservation legislation and management.

The distribution and abundance of *F. virsoides* on the Istrian coast has shrunk massively during the last 30 years, with some signs of regression mentioned in the literature as far back as the late 1960s and 1970s [4,5,11,20]. This may imply the abundance of the species may have been even larger prior to the 1960s and that the regression may have started earlier. However, the older literature data are floristic in nature (herbarium collections and author accounts), and they do not contain quantifiable information, only presence/absence and geographic location [3,15,17,18]. Photographic evidence of the species’ distribution is also very difficult to locate, with only a few usable examples. The implied reason for the regression of all fucalean algae since the 1960s was the ever-increasing pollution and eutrophication of coastal waters [20,21,22]. Fucalean biomass in general was presumed to be recovering in some localities during the late 1990s and early 2000s, with *F. virsoides* included [19,23,24]. However, while some fucalean species in the northern Adriatic were recovering well into the 2010s, *F. virsoides* regressed again, for example completely disappearing from the Slovenian coast [9,25]. 

More recent surveys from 2010–2016 have focused on specific parts of the coastline where the distribution was mapped, coverage was assessed, and biomass was measured, where possible [12,13,14]. These data were collected in order to be as comparable as possible with the data from Munda [5,11]. However, during 2016 surveys, destructive sampling (sample collection) for biomass assessment was abandoned due to low settlement density. 

**Figure 1:**
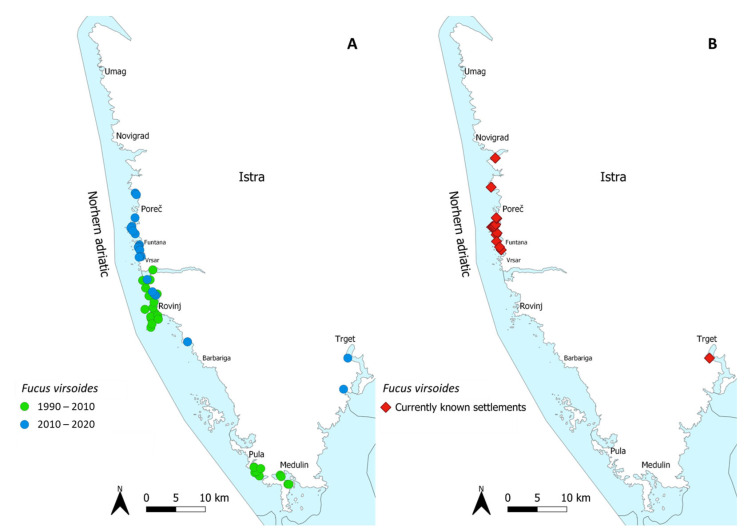
Historically known settlements of *Fucus virsoides* along the Istrian coast from 1990–2020 (**A**) and its current known settlements from mapping in 2021 (**B**).

Most recently, *F. virsoides* distribution was fully mapped along the Istrian coast during 2021, showing a further regression of the species with several settlements lost and others degraded [26]. Most of the surviving population on the Istrian coast is located in the vicinity of Poreč, Funtana, and Vrsar (Figure 1).

*F. virsoides* distribution is not well mapped in Croatia, and no recent data on its distribution exist along the neighbouring countries’ coastlines. Even though the distribution in Istria has been mostly mapped, for the rest of Croatia, the distribution of *F. virsoides* is still unknown, with only hearsay reports available, but no published data are available [27]. 

Since the remaining known settlements in Istria are small, isolated, and in severe regression, it is prudent to develop a method for enhancing or planting new settlements of *F. virsoides*. Here, we present the first ex situ experimental restoration of *F. virsoides* in Croatia. We tested whether *F. virsoides* can be grown ex situ in laboratory conditions and planted into a location previously hosting the species, reintroducing the species on a small scale in order to assess survivability. Furthermore, we also explored whether there are any differences in growth regarding the upper and lower positioning of planting plots in the intertidal zone.

## 2. Results

### 2.1. Early Growth

**Figure 2:**
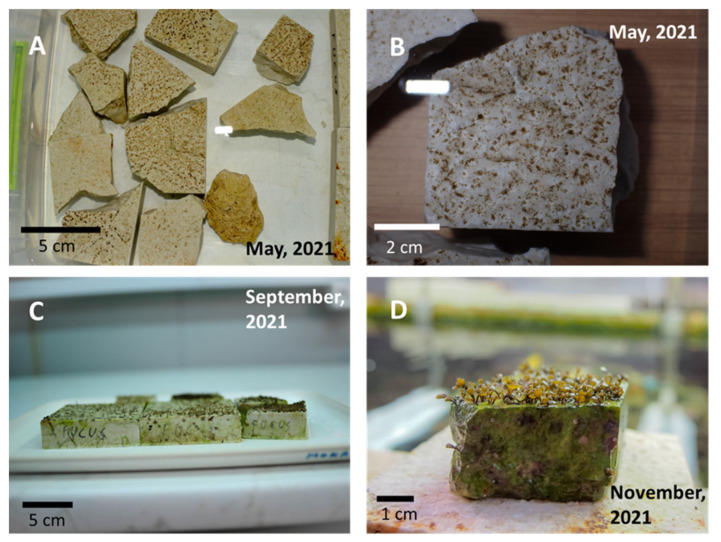
Early growth of *Fucus virsoides* recruits from May (**A**,**B**), September (**C**) and November 2021 (**D**).

Settled germlings became visible just a few hours after the seeding process started. Eight stone fragments (cca. 200 cm^2^ in total) were successfully seeded by germlings and retained recruits until the planting phase (Figure 2). Other stones were not densely seeded enough, and recruits did not manage to reach a suitable size before succumbing to fouling. The recruits effectively showed no growth for the first four months (May to September) and were too small to accurately measure. After they were moved to the outdoor system in September 2021 at the average size of 0.228 cm (±0.016 SE), an unexpected increase in average size up to 0.437 cm (±0.036 SE) was measured two months later in November 2021, just before planting (Figure 2D).

### 2.2. Post-Planting Growth

**Figure 3:**
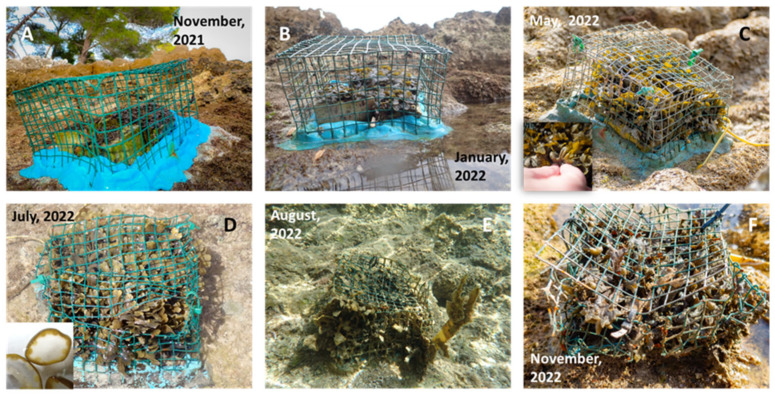
Post planting growth of *Fucus virsoides* over a one-year period. Photographs were taken in November 2021 (**A**), January 2022 (**B**), May 2022 (**C**), July 2022 (**D**), August 2022 (**E**) and November 2022 (**F**).

**Figure 4:**
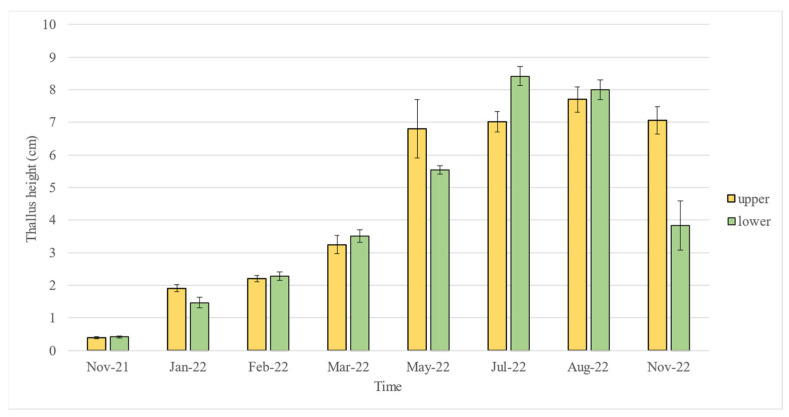
Growth of planted *F. virsoides* over a one-year period. Each measurement represents an average of 10 measured thalli. Data are presented as mean ± SE. Colours represent upper (yellow) and lower (green) positions for plots.

After planting *F. virsoides* in November 2021 in the designated positions, the growth accelerated dramatically (Figure 3). On the lower position, algae reached 1.470 cm (±0.159 SE) after two months, 3.520 cm (±0.193 SE) after five months, 5.540 cm (±0.135 SE) after seven months, and finally 8.420 cm (±0.283 SE) in height with fertile receptacles nine months post planting (Figure 4). On the upper position, algae reached 1.908 cm (±0.105 SE) after two months, 3.250 cm (±0.282 SE) after five months, 6.800 cm (±0.902 SE) after seven months, and finally 7.020 cm (±0.305 SE) nine months post planting (Figure 4). In August 2022 (10 months post planting), the growth stagnated at 8 cm (±0.301 SE) on the lower position, but it increased to 7.700 cm (±0.397 SE) on the upper position. In November 2022 (1 year post planting), a reduction in the average size of thalli was measured at 7.06 cm (±0.412 SE) on the upper as well as a strong reduction 3.840 cm (±0.753 SE) on the lower position. The average size of thalli across all measurement times is presented in Figure 4. 

**Figure 5:**
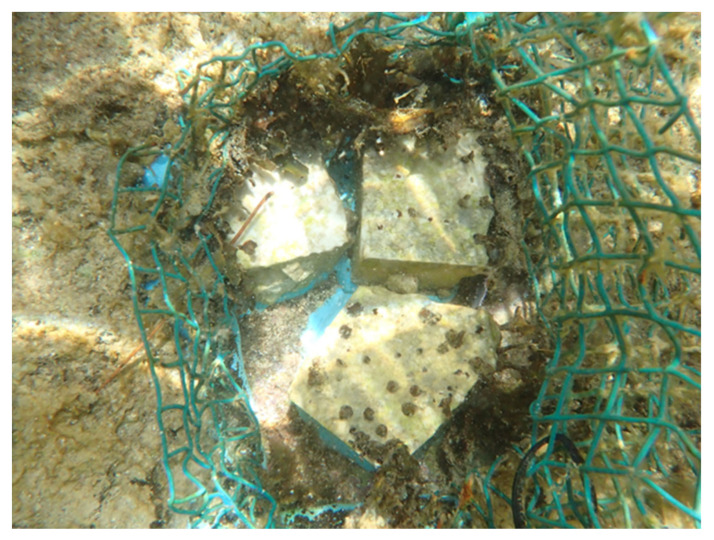
Destroyed anti-grazer cage with consumed *Fucus virsoides*.

In late August 2022, the protective cage on the lower position was destroyed by unknown means and fully grown, fertile thalli were exposed to grazers, which consumed most of the algal fronds, leaving only holdfasts (Figure 5). This destruction was the cause of such a strong decrease in the average height for the plots at the lower position in November 2022 (Figure 4). Interestingly though, some of the surviving specimens from the destroyed cage were still found to be fertile. 

### 2.3. Statistical Analyses

Two-way ANOVA and the SNK test for the “Position × Time” interaction showed that thallus length in May 2022 (seven months post planting) and November 2022 (one year post planting) was higher in the lower cage position than in the upper cage position. In July 2022 (after eight months), the thallus length was higher in the upper cage position, and no differences in thallus length between the upper and lower cage positions were found in all other times examined (Table 1). The difference between positions in November 2022 (one year post planting) can easily be explained by the destruction of the protective cage and overgrazing on the lower position in late August 2022 (after the August measurement). 

## 3. Discussion

While many fucalean algal species have been successfully grown ex situ, there has not yet been a successful and published attempt at growing *F. virsoides* for restoration purposes [28,29]. We mainly attribute the success of this restoration experiment to a high reproductive output of the source individuals.

Gametes were spontaneously released during the seeding process and thus did not require culturing or stress induction, as has been described elsewhere for a related species [30]. We did, however, observe during this experiment the increased and “explosive” release of gametes from the conceptacles when stress, such as intense light, desiccation, or temperature shock, was applied. The application of this was not considered necessary due to spontaneous gamete release in a sufficient quantity, possibly owing to the stress from transport. We also observed that *F. virsoides* thalli on the Istrian coast were fertile all year round, which is a contradiction to published works, suggesting that the species is only fertile in the spring [3]. Despite the many possibilities of growth enhancement, this method has been kept as simple as possible, with reproducibility in mind. We have successfully replicated this method and planted two more plots during 2022 in the same area and one more plot in a nearby area afterwards. However, these were not monitored in the same way, and therefore the results are not included.

The results presented here suggest that there are some significant differences in average thalli height at different positions at certain measurement times. While the difference in November 2022 is evidently due to overgrazing since the protective cage was destroyed on the lower position, other differences (in May and July 2022) are difficult to determine. This, however, is not of major importance for the reintroduction and conservation of *F. virsoides,* since algae at both positions reached fertility at the same time, with only minor differences in thallus height.

**Figure 6:**
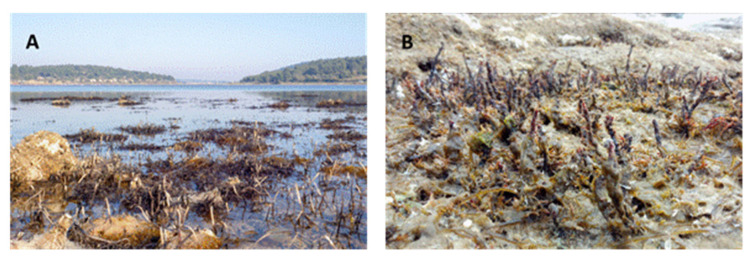
Fucalean species damaged during springtime low water-high air temperature event on southern (**A**) and western (**B**) Istrian coast.

*F. virsoides* is found in the intertidal zone of coastal marine systems and thus must adapt to extreme biotic and abiotic environmental factors. So far, the effects of most of these factors on the growth and development of *F. virsoides* have not been assessed in a rigorous way or were merely discussed within the previous literature. We hypothesize that emersion can have positive effects for *F. virsoides*, and substrate anti-fouling may reduce competition for space and nutrients. Most importantly, periods of emersion may offer direct relief from grazing pressure and increase survivability. Specifically, the fish *Sarpa salpa* (Linnaeus, 1758) and urchin *Paracentrotus lividus* (Lamarck, 1816) are the dominant herbivores in the Adriatic, and they readily feed on all fucalean algae, but they find *F. virsoides* hard to reach during the low tide [31,32]. Therefore, emersion periods may be very important for the species’ growth, development, and survivability. However, if “spring tides” coincide with extreme heat waves, leaving the settlements of *F. virsoides* exposed to hot and dry conditions may cause heavy damage to thalli and settlement as a whole. Such events will, however, only occur during winter and spring seasons, when low and minimum water levels are reached during daytime. These effects have been observed before on other species in California [33] and more locally, on *Ericaria* and *Gongolaria* species in the intertidal zone of the southern and western Istrian coast (author’s unpublished data, Figure 6).

Temperature is the main driver of metabolism, growth, productivity, and respiration for the whole organism [34]. It also controls the solubility and availability of gases. *F. virsoides* inhabits the intertidal zone and is able to survive extremely high and low temperatures, but it also exhibits two distinct lifestyles; one as a terrestrial “plant”, fully exposed to sunlight, and the second as a submerged “plant”, protected from direct sunlight. If specimens are emersed during the day (especially during hot days), the thalli can suffer certain damage due to high temperature and intense sunlight, as well as desiccation [35,36]. This is, however, mostly mitigated by the species’ regeneration abilities, as well as naturally thick settlements with larger, more robust specimens, keeping the lower canopies moist. If the emersion occurs during the night, this can be averted.

Salinity was found by Vouk [37] and Linardić [3] to be an important factor in *F. virsoides* distribution. Recently, Orlando Bonaca et al. [8] correlated the abundance of *F. virsoides* on Slovenian and Italian coastline (Gulf of Trieste) with the presence of freshwater sources or fluctuating salinity. This conclusion is confirmed in both historical and more recent distribution data [3,5,19]. Other members of the genus *Fucus* are indeed typically found in areas of lower salinity than the *F. virsoides*, although this could be only due to geographic isolation and subsequent speciation [38]. During ex situ development, salinity was regulated only by regular water changes and was as such not considered a major factor for growth. The full effect of fluctuating salinity on the growth and fitness of *F. virsoides* requires a dedicated experimental approach.

*F. virsoides* is typically found in semi-exposed or semi-sheltered areas with low sloping coastline in the mid-tidal zone [4,5,11,19,20]. Orlando Bonaca et al. [8] also correlated the distribution and abundance of the species to the stability of substrata and wave exposure, while Lipizer [39] noticed the relation with the wind exposure. The type of substrate is also very important, since it defines the longevity and stability of the attachment surface for the algal growth [40,41,42]. While *F. virsoides* will attach and grow on any solid and fixed substrata, it is important to consider this information when planning a planting experiment, otherwise the cultivated specimens could suffer mechanical damage from abrasion from coarse sediment. 

*F. virsoides* can easily be considered as strongly photophilic species due to its typical habitat [3]. This, however, might not be the best representation, since the alga has evolved to resist strong sunlight exposure during emersion, not to exploit it per se. While there is data to confirm this, the topic still requires more refined research [36].The species can be found both in sunlight-exposed and shaded coastal areas. The coastal areas of the Istrian peninsula have been artificially or naturally re-forested during the last 70 years, mostly due to abandonment of agricultural practices near the coast in favour of tourism, as well as natural succession. This could have potentially “shaded” the *F. virsoides* settlements and affected their distribution and abundance on the local scale, but nowadays we can only speculate due to the severely diminished presence of *F. virsoides.* The disappearance of *F. virsoides* from most of the semi-urbanised coastline areas is also difficult to explain, since most of the remaining settlements are, in fact, found near tourist infrastructure. During the laboratory cultivation of *F. virsoides* in our study, LED lights used for the first five months have shown very little effect on the growth of the recruits. When moved to outer tanks with natural seawater at ambient temperature and fluorescent tube lighting, the growth markedly accelerated. While this cannot be fully attributed to the lighting conditions, it remains a distinct possibility, since many ex situ growth methods used fluorescent tube lights instead of LED, or we may have used an unsuitable combination of lightning factors (wavelength and intensity).

No reduction in the cover of *F. virsoides* was noticed from the time of planting in November until late August (after nine months), since the plots were completely covered with fronds that extended through the protective cage meshes (Figure 3). While the measurements were originally planned as quadruplicates per plot, the cover was not discernible after the second measurement due to overcrowding of *F. virsoides* thalli, and therefore no meaningful results could be presented. The anti-grazing cages have proven very effective at protecting the planted thalli. However, they were often damaged due to external factors (wave action, pebbles, stones, trampling) and needed regular repairs. During the experiment, the cage on the lower position was destroyed, which allowed the grazers to consume most of the available thalli inside (Figure 5). This was, in fact, the only measured reduction in cover during the experiment. The presumed culprit, *Sarpa salpa,* can often be observed browsing the intertidal zone during high water level and is a predominant Mediterranean and Adriatic herbivorous fish species [43,44]. While grazing pressure is expected and can often drive succession as well as community structure, during longer timeframes, plants (or algae) with extremely small populations (or PSESP-s) are extremely susceptible to overgrazing, as this could further diminish, prevent recovery, or even extirpate the populations [45,46,47]. While protecting these small settlements can prevent overgrazing, if such an experiment were to be replicated on a larger scale, some simpler method of grazer exclusion must be developed, or an optimal point in relation to the mean sea level should be identified in order for the macro-grazers to be effectively excluded. Furthermore, rock pools above the mid tide level could possibly be artificially planted or seeded with *F. virsoides* in order to establish a kind of “refugia” for this species. This phenomenon, of fucalean algae retreating into the rockpools, outside of the reach of grazers, has been regularly observed along the eastern Adriatic coast and in this particular case, which may be used to artificially establish long-term refugia, despite rockpools not being the most common habitat for *F. virsoides*.

## 4. Materials and Methods

### 4.1. Seeding

**Figure 7:**
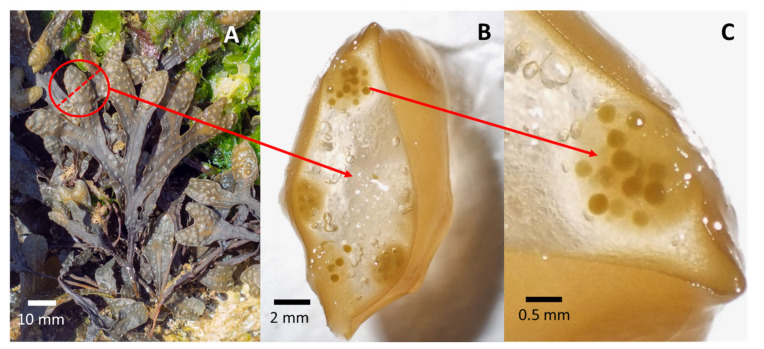
*Fucus virsoides* thallus with receptacle marked with a red circle (**A**), transverse section of a receptacle showing conceptacles (**B**), magnification showing conceptacle with mature gametangia (**C**).

**Figure 8:**
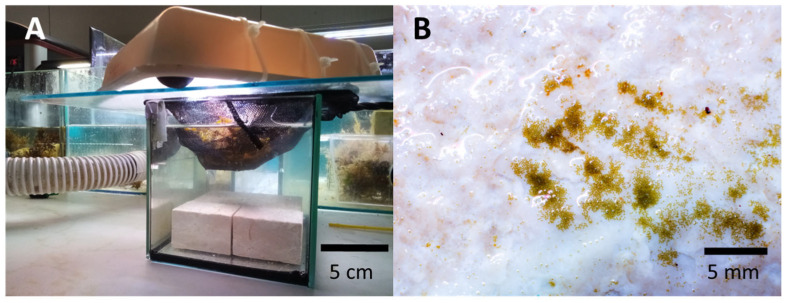
Seeding of *Fucus virsoides* onto limestone tiles (**A**) and early stage germlings (**B**).

The fertile receptacles were collected by hand from a surviving settlement in Funtana, near Poreč—Western Istrian coast on 23 April 2021 (Figure 1). Receptacles were checked for fertility in situ by cutting a transverse section of the receptacle either by a razorblade or by a tip of a fingernail. Afterwards, the thick conceptacles with female gametes were clearly observable using a camera with a larger than 1:1 macro capability (Olympus Tough TG-6) (Figure 7A–C). While both male and female gametes are produced in the same receptacle, the male gametes are only observable under high magnification of a microscope (Olympus SZX 12). A small aquarium filled with unfiltered natural seawater (5 L) was used to hold fragmented limestone tiles (i.e., stones) of a similar size (Figure 8). A small net was filled with cca. 30 fertile receptacles from multiple individuals and placed in the aquarium on the water surface for the fertilization to happen in the water and the zygotes to settle on the stones (Figure 8). Aeration and mixing were performed by an air pump.

### 4.2. Early Growth

After germlings became visible (1 week), stones were moved to a bigger, 24 L closed aquarium system with controlled temperature and light (at 16–18 °C, LED GNC SilverMoon Marine, 148 μmol photons m^−2^ s^−1^, 12 h light/dark period). Full water changes were also performed weekly. Stones were photographed in order for the height of the germlings to be measured using ImageJ [48]. Unfortunately, this was not successful during the early growth period due to highly unreliable results. However, in September 2021, when germlings became more prominent and measurable, seeded stones were moved to outer open-flow stone basins with fluorescent tube lightning (Phillips Master TL-D 36 W/865, 6000 K, 95 μmol photons m^−2^ s^−1^) in the Center for Marine Research courtyard in order to accelerate their growth. These basins (1300 L) have a constant flow of natural unfiltered seawater pumped from the nearby bay. From then until planting (two months), *F. virsoides* recruits were kept under those conditions, and 20 random specimens were used in order to assess the average size of thalli and the standard error. Light intensity and temperature were measured during the process by Hobo Pendant MX Temperature/Light Data Logger logged every 60 min in order to monitor the conditions.

### 4.3. Planting

**Figure 9:**
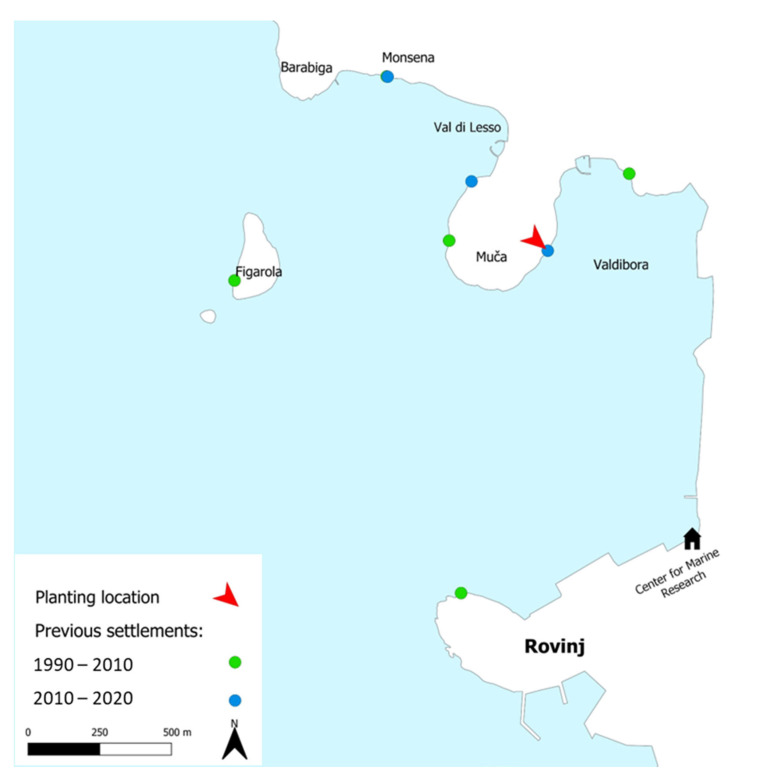
Past distribution of *Fucus virsoides* settlements in the Rovinj area. Area of planting is shown with a red mark.

The recruited individuals were planted in Muča, Rovinj, which previously hosted *F. virsoides* settlements (Figure 9). The previous settlement was marked with a blue epoxy in 2014 [13], and planting was performed by attaching the stones by the same epoxy (Sub Coat XT Azzuro 2/1, Veneziani) within the vicinity of these markings. Plots were covered by mesh cages (2 plots, 100 cm^2^, 4 stones per plot, covered with protective cage 12 × 12 × 10 cm in size, mesh opening of 1 cm) in order to protect the recruits from potential grazers (such as limpets and other gastropods, hermit crabs, *Sarpa salpa,* and urchins). Each of the two cages were placed on approximately the upper and lower edges of the mid-tidal zone (~3 m apart) in order to assess if positioning of the plots can significantly affect the growth and survivability. The cages were repaired when damaged and cleaned, as necessary, from fouling. Percent cover was monitored via photography, thallus height was measured manually with a ruler, and timing of fertility was monitored in situ. This was performed during the next 12 months. In each plot, 10 random recruits (and later juveniles and adults) were used in order to measure the average growth and standard error.

### 4.4. Statistical Analysis

The thalli length of *F. virsoides* was analysed by two-way ANOVA using “Position” (2 levels: Upper and Lower) and “Time” (8 levels: November 2021, January 2022, February 2022, March 2022, May 2022, July 2022 August 2022, November 2022) as fixed factors. Prior to ANOVA, data were tested for homogeneity of variance using Cochran’s C-test. The significant “Position × Time” (*p* < 0.003) interaction was analysed by post hoc Student-Newman-Keuls (SNK) tests. Analysis of variance (ANOVA) was performed by GMAV-5 for Windows.

This study was performed in accordance with the permission by the Croatian Ministry of Environmental and Nature Protection, Department for Environmental and Nature Protection (Ministarstvo gospodarstva i održivog razvoja, Zavod za zaštitu okoliša i prirode).

## 5. Conclusions

While *F. virsoides* remains on the brink of regional extinction on the Istrian coast, we showed that ex situ cultivation and planting of this species using a simple method is possible, at least at the small-scale. This was primarily successful due to the unpredictably fast growth of the *F. virsoides* after the planting and the resilience of the grown thalli. Even though the growth of the planted *F. virsoides* showed some differences according to the positioning in the intertidal zone, this does not present an obstacle for the possible further restoration attempts on the Istrian coast. Further research pertaining to the interactions of this species within its restored environment will help inform best practices for scaling up restoration techniques with the goal of re-establishing new settlements in the future.

## Figures and Tables

**Table 1:** Two-way ANOVA testing the effects of two positions of *Fucus virsoides* in the intertidal (upper and lower) on thallus growth.

Source	df	MS	F	*p*
Position	1	2.4337	0.38	0.5556
Time	7	160.2287	104.77	0.0000
Position × Time	7	6.3555	4.16	0.0003
Residual	144	1.5293		
Cochran’s test: 0.33, *p* < 0.05				
SNK test for the interaction Position x Time:				
November 2021: Upper = Lower January 2022: Upper = Lower February 2022: Upper = Lower March 2022: Upper = Lower		May 2022: Upper > Lower July 2022: Upper < Lower August 2022: Upper = Lower November 2022: Upper > Lower		

Factors: Position (fixed, 2 levels—Upper and Lower), Time (random, 8 levels—November 2021, January 2022, February 2022, March 2022, May 22, July 2022, August 2022, November 2022). Number of replicates per each combination of factor levels *n* = 10. Total number of replicates *n* = 160.

## Data Availability

The original contributions presented in the study are included in the article. Further inquiries can be directed to the corresponding author.
